# PET/CT radiomics and deep learning in the diagnosis of benign and malignant pulmonary nodules: progress and challenges

**DOI:** 10.3389/fonc.2024.1491762

**Published:** 2024-11-08

**Authors:** Yan Sun, Xinyu Ge, Rong Niu, Jianxiong Gao, Yunmei Shi, Xiaoliang Shao, Yuetao Wang, Xiaonan Shao

**Affiliations:** ^1^ Department of Nuclear Medicine, the Third Affiliated Hospital of Soochow University, Changzhou, China; ^2^ Institute of Clinical Translation of Nuclear Medicine and Molecular Imaging, Soochow University, Changzhou, China; ^3^ Department of Nuclear Medicine, Changzhou Clinical Medical Center, Changzhou, China

**Keywords:** pulmonary nodules, lung neoplasms, PET/CT, radiomics, deep learning

## Abstract

Lung cancer is currently the leading cause of cancer-related deaths, and early diagnosis and screening can significantly reduce its mortality rate. Since some early-stage lung cancers lack obvious clinical symptoms and only present as pulmonary nodules (PNs) in imaging examinations, accurately determining the benign or malignant nature of PNs is crucial for improving patient survival rates. ^18^F-FDG PET/CT is important in diagnosing PNs, but its specificity needs improvement. Radiomics can provide information beyond traditional visual assessment, overcoming its limitations by extracting high-throughput quantitative features from medical images. Radiomics features based on ^18^F-FDG PET/CT and deep learning methods have shown great potential in the noninvasive diagnosis of PNs. This paper reviews the latest advancements in these methods and discusses their contributions to improving diagnostic accuracy and the challenges they face.

## 1 Introduction

According to the 2024 statistics from the American Cancer Society, lung cancer is one of the leading causes of cancer-related deaths (1). About 50% of patients are already at a locally advanced or distant metastasis stage at the time of diagnosis, thus missing the optimal window for surgical treatment (2). However, early-stage lung cancer often lacks obvious clinical symptoms and typically only presents as pulmonary nodules (PNs) in imaging examinations, with approximately 35% of solitary pulmonary nodules (SPNs) being diagnosed as early-stage primary lung cancer (3). Thus, accurately diagnosing the benign or malignant nature of PNs is key to improving patient prognosis and survival rates.

Although biopsy is considered the ‘gold standard’ for confirming the benign or malignant nature of PNs, it has several limitations, such as being highly invasive, having poor repeatability, carrying a high risk of complications, and being unable to provide whole-body assessment or spatial information for non-puncture sites (4). Although traditional CT has some advantages in non-invasive screening of PNs, its diagnosis mainly depends on anatomical information. Therefore, it may show false positives in some cases, especially in overdiagnosing some benign lesions (such as granulomas and calcifications). And a report (5) shows that as many as 50% of the nodules removed during surgery are benign, indicating that it is difficult for radiologists to determine whether a pulmonary nodule is malignant based on CT and other clinical information (6). Currently, dual-modality ^18^F-FDG PET/CT imaging, which combines the anatomical information of CT and the metabolic information of PET, is widely used in diagnosing PNs, and its diagnostic accuracy is superior to that of PET or CT alone (7–11). However, ^18^F-FDG PET/CT still faces the challenge of a high false-positive rate when diagnosing the benign or malignant nature of PNs (12–15). This issue often arises because certain benign lesions (such as granulomas, organizing pneumonia, or fungal infections) are difficult to distinguish from malignant PNs based on imaging features (12, 16). Although ^18^F-FDG PET/CT has a high sensitivity (SEN: 0.89) in detecting malignant SPNs, its specificity is relatively low (SPE: 0.70) (17). Therefore, further research is needed to improve the accuracy of PNs diagnosis.

**Figure 1:**
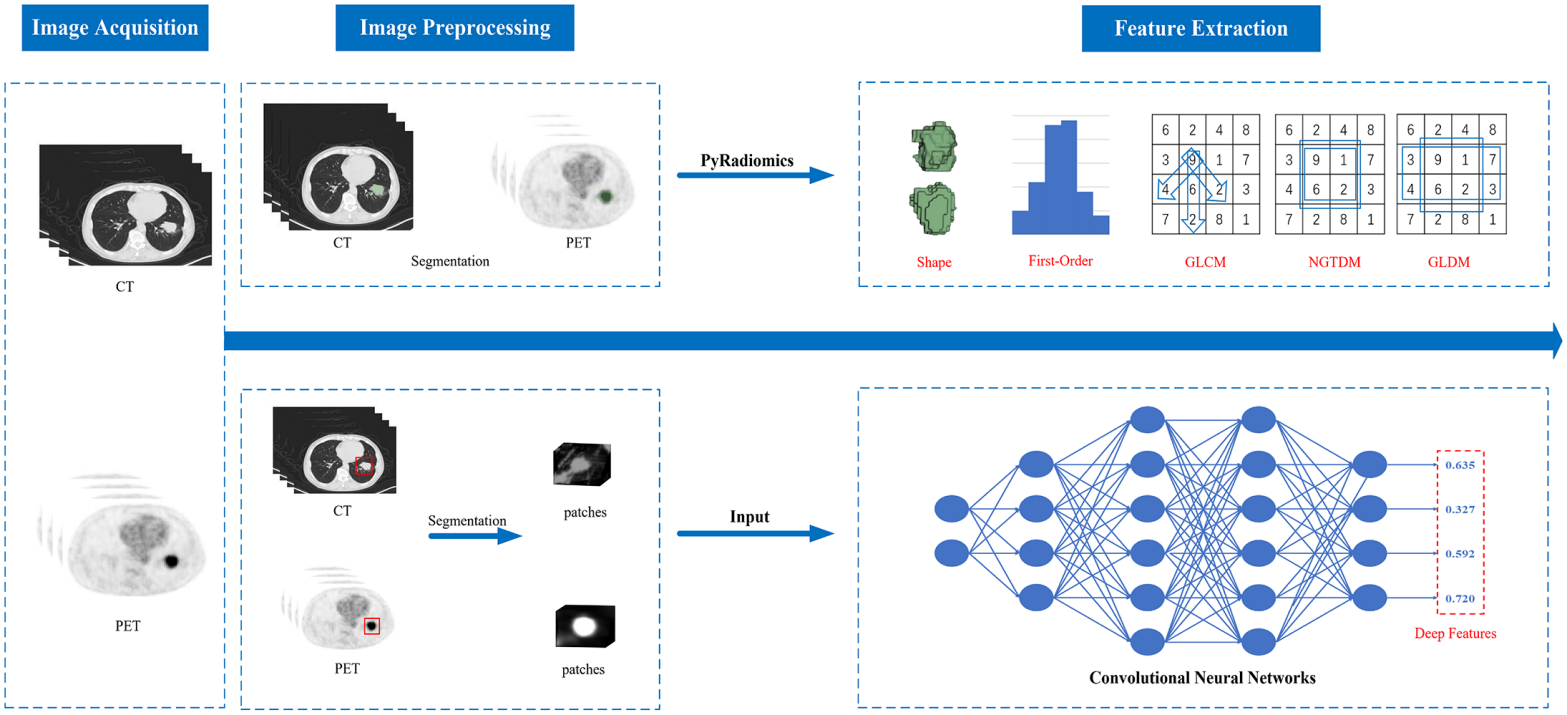
The differences in procedures between extracting handcrafted features and deep features.

In recent years, radiomics has been an emerging medical image analysis technology. This concept was first put forward by the Dutch scholar Lambin (18) in 2012. Radiomics generally refers to the high-throughput extraction of many quantitative features from medical images (such as CT, MRI, PET, etc.) and converting the image data of the region of interest into mineable high-dimensional data (19). Its features include intensity, shape, volume, texture features, etc. (20), which can effectively address the limitations of traditional assessment methods and offer new perspectives for diagnosing benign and malignant PNs with ^18^F-FDG PET/CT (21–25). Radiomics features are divided into handcrafted features (HF) and deep features (DF) according to different extraction methods. **Figure 1** illustrates the differences in procedures between extracting handcrafted features and deep features. Handcrafted features are predefined or manually extracted by image processing experts, while deep learning (DL) algorithms automatically extract deep features without human intervention (26, 27). In recent years, both HF and DF have been widely used in tasks such as image classification and regression, demonstrating great potential in improving the performance of diagnostic models. However, effectively combining these features in diagnosing PNs and addressing the challenges that arise from this combination remain key areas of current research. This paper reviews the methodological advancements of ^18^F-FDG PET/CT radiomics and deep learning in diagnosing benign and malignant PNs, focusing on the application and challenges of HF and DF in enhancing diagnostic model performance.

## 2 Application of HF in the diagnosis of benign and malignant PNs

Handcrafted features (HF) extracted by traditional radiomics can generally be divided into four categories: statistical features (such as histogram-based features and texture features), model-based features, transform-based features, and shape-based features (28).

Histogram-based features include the mean, maximum, minimum, variance, and percentiles of gray levels (28, 29). These features are typically based on single-pixel or single-voxel analysis and thus are referred to as first-order features. Texture features, also termed second-order characteristics, involve entities such as the Gray-Level Co-occurrence Matrix (GLCM) and the Neighborhood Gray-Tone Difference Matrix (NGTDM), which are employed to depict the interrelationships among voxels. Model-based features characterize the features of an object or shape by analyzing the gray-level information in space. Transform-based features are extracted through methods like Fourier transform, Gabor transform, or Haar wavelet transform and are utilized to analyze gray-level patterns in different spaces (19). Shape-based features, such as compactness and sphericity, depict the geometric attributes of the region of interest (ROI).

### 2.1 Differentiating diagnosis of PNs using HF alone in PET/CT imaging

Multiple studies have evaluated the ability of handcrafted features (HF) from ^18^F-FDG PET/CT to differentiate between benign and malignant PNs (see **Table 1**) (22, 30–32). For example, Liu et al. (30) studied 20 CT radiomics features, including 14 gray-level co-occurrence matrix (GLCM) features, 1 intensity histogram feature, and 5 shape features, to distinguish between peripheral lung cancer and inflammatory pseudotumor. They found that GLCM features had the highest discriminative ability. Additionally, Chen et al. (22) extracted neighborhood gray-tone difference matrix (NGTDM) features from dual-time-point imaging (DTPI) ^18^F-FDG PET/CT images and found that these features had an AUC of 0.89 in diagnosing benign and malignant solitary pulmonary nodules (SPNs), outperforming the maximum standardized uptake value (SUVmax, AUC: 0.75) or visual assessment (AUC: 0.80). This finding is consistent with the results of Bomhals et al. (31). Texture features, as part of HF, capture tissue structural heterogeneity by quantifying the relationship between voxels and their surroundings, thus performing well in the diagnosis of benign and malignant PNs. However, using HF alone may be insufficient to fully describe tumor heterogeneity (32). Combining HF with other features, such as CT semantic features, PET metabolic parameters, and clinical features, can significantly improve the accuracy and reproducibility of ^18^F-FDG PET/CT radiomics in diagnosing benign and malignant PNs (33).

**Table 1:** Summary of literature on the diagnosis of benign and malignant lung nodules based on HF.

Author	Year	Case number (case)	Benign nodules (number)	Malignant nodules (number)	Extracted radiomic features	Classifier	Model Evaluation (models)	Best model AUC/ACC
Chen et al. (22)	2019	116	25	81	3	NA	5	Visual analysis with texture features AUC: 0.890
Kang et al. (34)	2019	268	111	157	4338	LASSO, LR	5	Hybrid nomogram (PET/CT RS + manual diagnosis) AUC: 0.980
Teramoto et al. (38)	2019	33	18	18	25	RF	3	Classification based on CT images and early and delayed PET images AUC: 0.895
Zhang et al. (23)	2020	82	37	45	102	SVM, LASSO, LR	3	SVM model AUC: 0.854
Liu et al. (30)	2020	42	21	21	435	LR	20	5 shape features AUC: 0.748
Palumbo et al. (24)	2020	111	39	72	18	ClT, KNN, NB	24	NB that combines PET traditional imaging features, shape and texture features ACC: 0.824
Hu et al. (25)	2021	235	104	131	92	LR, LASSO	3	Complex model (PET/CT imaging model + clinical model) AUC: 0.909
Niu et al. (35)	2021	165	23	167	49	LR, LASSO	5	PET+CT (1mm) imaging model AUC: 0.940
Albano et al. (32)	2021	202	64	127	42	LR	3	Combining GLCM and GLZLM features AUC: 0.861
Ren et al. (40)	2022	280	128	152	157	LASSO	3	Combined model (radiomic model + clinical model) AUC: 0.940
Agüloğlu et al. (36)	2023	106	53	53	41	LR, NB, SVM, KNN, J48, RF	6	LR classification model AUC: 0.813
Wang et al. (41)	2023	1068	356	712	12	LR, OPLS-DA	3	Machine Learning Model AUC: 0.890
Ning et al. (37)	2023	113	63	60	678	LR, LASSO,	2	^18^F-FDG PET/CT+FLT features AUC:0.879
Elia et al. (39)	2023	71	25	46	71	Functional Tree, Rep Tree, J48	3	J48 AUC: 0.932
Bomhals et al. (31)	2023	39	20	19	118	LR	2	LR model with two PCs AUC: 0.770
Zheng et al. (10)	2024	190	69	121	396	LR, LASSO	3	PET/CT model AUC: 0.929

NA, not Available; NB, naive bayes; RF, random forest; RS, radiomics signatures; SVM, support vector machine; LR, logistic regression; LASSO, least absolute shrinkage and selection operator; ClT, classification tree; KNN, k-Nearest Neighbors; J48, decision tree; OPLS-DA, orthogonal partial least squares discrimination analysis; PCs, principal components; ACC, Accuracy; AUC, area under curve.

### 2.2 Differentiating diagnosis of PNs using a combination of HF and other features in PET/CT imaging

Since benign lesions (such as inflammatory lesions) and malignant tumors have significant differences in biological behavior, pathological processes, and internal structures, combining the complementary nature of various features can provide a more comprehensive description of the benign and malignant differences in PNs, leading to more accurate qualitative diagnoses. **Table 1** summarizes 16 studies that utilized HF for the diagnosis of benign and malignant lung nodules, of which 15 studies combined PET/CT imaging HF with other features (such as CT semantic features, PET metabolic parameters, and clinical features) for diagnosis (10, 22–25, 31, 32, 34–41). These studies reported diagnostic AUC values ranging from 0.813 to 0.980, demonstrating the great potential of combining HF with other features to improve model performance.

In clinical diagnosis, physicians typically rely on the CT semantic features of PNs (such as morphology, density, internal structure, etc.) to determine their nature. Although these features play an important role in diagnosis, their accuracy may be reduced due to the influence of subjective factors (35). In contrast, HF from PET/CT can provide objective quantitative information that is not obtainable through traditional visual assessments. Studies have shown that combining HF with CT semantic features can more effectively differentiate between benign and malignant PNs, with diagnostic AUC values ranging from 0.875 to 0.980 (22, 34, 35). For example, Kang et al. (34) extracted features from CT, thin-slice CT, PET, and PET/CT images, used the LASSO algorithm to select the most significant features and calculated a radiomics score (RS), which was then combined with manual diagnosis to construct a hybrid nomogram. The results showed that the AUC of the hybrid nomogram exceeded 0.89 and significantly reduced the false positive rate (FPR) from 30.9% in manual diagnosis to 9.1%. Additionally, studies (35) have shown that a model combining CT semantic features with radiomics features has significantly better diagnostic performance than a model using RS alone (AUC: 0.908 vs. 0.704) while substantially reducing the FPR. These findings indicate that combining radiomics with CT semantic features can significantly improve the accuracy of PNs diagnosis.

Studies have also shown that combining HF from PET/CT with PET metabolic parameters can significantly enhance the diagnostic performance for differentiating between benign and malignant PNs (22–24, 35, 36, 41). For example, Palumbo et al. (24) retrospectively evaluated data from 111 patients and found that combining PET and CT shape and texture features with PET conventional metabolic parameters could improve the diagnostic accuracy of the model by 2.2% to 10.2%. Zhang et al. (23) analyzed PET/CT images from 82 patients and compared the diagnostic performance of a support vector machine (SVM) model based on SUVmax, metabolic tumor volume (MTV), and texture features, finding that the AUC of the SVM model was 0.854, which was superior to using only the SUVmax (AUC: 0.595) or MTV model (AUC: 0.616). Niu et al. (35) significantly improved the diagnostic performance by incorporating SUVmax into a CT radiomics model, with the AUC increasing from 0.704 to 0.940. These results further support the idea that combining radiomics features with PET metabolic parameters can enhance the diagnostic capability of the model.

Additionally, studies have explored strategies to improve the qualitative diagnosis of PNs by combining HF from PET/CT with clinical features (25, 40, 41). This fusion strategy is divided into pre-fusion and post-fusion (see **Table 2**). Pre-fusion involves merging the selected radiomics features with clinical features before model training. Wang et al. (41) studied PET/CT data from 187 patients with non-small cell lung cancer (NSCLC) and 190 patients with benign lung nodules, incorporating clinical features such as gender, age, and smoking history. The results showed that the model’s AUC reached 0.890, significantly improving the diagnostic ability of early SPNs. Conversely, post-fusion involves independently constructing HF and clinical feature models and combining them to form a complex model. Ren et al. (40) developed clinical, radiomics, and combined models using data from 280 patients. The results showed that the combined model exhibited the best AUC (0.910 and 0.940) and the lowest false positive rate (FPR: 18.68% and 5.41%) in the classification of solid lung nodules. However, the study by Hu et al. (25) found that although the complex model combining radiomics features and clinical features had a slightly higher AUC than the radiomics model alone in distinguishing solitary pulmonary adenocarcinoma from pulmonary tuberculosis, the difference was not significant (training set AUC: 0.884 vs. 0.861; validation set AUC: 0.909 vs. 0.889). This may be related to the impact of different scanners on the accuracy of radiomics features (32). Therefore, whether combining clinical features with radiomics features can significantly improve diagnostic performance still requires further research.

**Table 2:** Comparison of pre-fusion and post-fusion strategies.

	Pre-fusion	Post-fusion
Fusion level	Data layer fusion	Decision-making layer fusion
Concept	In the early stages of feature processing, data or features from different sources (such as radiomics and clinical data) are combined.	Integrate outputs from different models (e.g., radiomics and clinical models) at a later stage of model training or after model training is complete.
Advantages	Allows to capture interactions between data early in model training, potentially improving the overall predictive power of the model	Allows specialized processing and optimization for each data type, increasing model flexibility and interpretability
Disadvantages	Need to solve the compatibility issues between different data sources to ensure data consistency and standardization.	It may not fully capture potential interactions between different data sources, which can sometimes reduce the power of combined forecasts.

Although HF is beneficial for reducing the false-positive rate of PET/CT in diagnosing pulmonary nodules, there are three major issues in extracting HF. First, image segmentation often relies on manual delineation, a process that is not only time-consuming but also prone to consistency issues between different operators and within the same operator at other times, thereby affecting the stability of the results. Second, the variability in the selection and processing of various image features can lead to inconsistencies in the analysis results, making it difficult to verify accuracy and reproducibility, increasing the likelihood of computational errors, and heightening diagnostic uncertainty. Lastly, although manual methods can extract various features, they are limited in their ability to comprehensively capture all the information within the Region of Interest (ROI), potentially missing details critical to the diagnosis (42). These limitations have driven the increasingly widespread application of DF. Deep features are automatically identified and extracted by deep learning algorithms, reducing human intervention and enhancing the accuracy and efficiency of analysis.

## 3 Application of DF in the diagnosis of benign and malignant PNs

DF is extracted from medical images through deep neural networks, particularly Convolutional Neural Networks (CNN). Compared to HF, DF can capture more complex and higher-level features (42). The extraction process of DF does not rely on manual operations, reducing human errors and significantly improving the accuracy of image data analysis. By automatically learning and identifying complex patterns within the data, DF enhances features’ representational power and substantially increases diagnoses’ accuracy and reliability. **Table 3** summarizes five studies that used DF to diagnose benign and malignant pulmonary nodules (11, 43–46).

**Table 3:** Summary of Literature on the diagnosis of benign and malignant pulmonary nodules based on DF.

Author	Year	Patient (case)	Benign nodules (number)	Malignant nodules (case)	Model	Model Evaluation (models)	Best model AUC/ACC
Park et al. (43)	2021	359	102	257	ResNet-18	8	PET/CT radiomics model with inclusion of SUVmax and lesion size AUC: 0.877
Shao et al. (11)	2021	106	23	92	3D-CNN, LR	3	PET/CT 3D-CNN AUC:0.970; PET 3D-CNN AUC:0.970
Lai et al. (44)	2022	112	33	79	HRNet, ResNet	4	Manual HRNet AUC: 0.789
Zhang et al. (45)	2023	174	77	97	RF, ResNet, DCNN	4	Combined Model AUC: 0.840
Alves et al. (46)	2024	113	62	51	CNN	7	Stacked 3D CNN model AUC: 0.839

ResNet, residual network; 3D-CNN, three-dimensional convolutional neural network; LR, logistic regression; HR Net, High-Resolution Network; RF, random forest; DCNN, deep convolutional neural networks; CNN, convolutional neural network; ACC, Accuracy; AUC, area under curve.

Deep Learning (DL) utilizes multi-layer feedforward neural networks to directly receive image inputs and perform end-to-end training in a supervised environment, thereby learning highly discriminative image features. The DL modeling includes image acquisition, preprocessing, model training, and analysis (47). In diagnosing benign and malignant Pulmonary Nodules (PNs), in addition to traditional 2D models, 2.5D and 3D models have also been developed. The 2.5D models input coronal, sagittal, and axial 2D images into three channels for modeling (48), while the 3D models integrate the continuous scan layers of pulmonary nodules into a three-dimensional object, further enhancing the ability to extract stereoscopic information (49). For instance, Alves et al. (46) used a 3D-CNN architecture model, and although only PET images were input, its diagnostic performance surpassed that of the 2D ResNet-50 model (AUC: 0.839 vs 0.774). Lai et al. (44) proposed a 3D High-Resolution Network (3D HRNet) architecture, which avoids stride layers and pooling layers to maintain the original dimensions of features, showing higher diagnostic accuracy than ResNet (AUC: 0.781 vs 0.650). Although 3D models generally outperform 2D models, in some cases, 2.5D models may perform more accurately (48), possibly due to overfitting issues in 3D models.

Deep learning-based radiomics (DLR) combines deep learning (DL) and traditional radiomics. While handcrafted features (HF) struggle to express tumor heterogeneity fully, limiting the performance of diagnostic models, DLR compensates for this by extracting deeper and higher-dimensional features through convolutional kernels, more comprehensively quantifying tumor heterogeneity (50). Studies show that combining HF and deep features (DF) can significantly improve model performance (45). For instance, Zhang et al. (45) extracted 100 HF and 2048 DF obtained through ResNet from PET and CT images to distinguish tuberculosis nodules from lung cancer. The results indicated that the deep convolutional neural network model with both HF and DF input (Radiomics-DCNN, AUC: 0.820) outperformed the DL model with only DF input (AUC: 0.720) and the traditional machine learning model with only HF input (AUC: 0.760). Although DLR has shown its potential, research in this field is still scarce, and further studies are needed to validate its feasibility and performance.

Although HF and DF employ different methods in radiomics, their goals and application scenarios are similar. With advancements in deep learning (DL) technology, DF typically outperforms HF in diagnostic performance, provided there is sufficient training data (see **Table 4** for specific comparisons (42, 51). HF is susceptible to variations in manual segmentation and scanning parameters, whereas DL models improve robustness and generalization by organically integrating feature extraction and classification through end-to-end training (52). However, DF has weaker interpretability, requires more data, and incurs higher computational costs. Therefore, HF still holds value in situations with limited data or specific tasks.

**Table 4:** Comparison between HF and DF.

	HF	DF
Feature extraction method	Extract features from images through manually defined algorithms based on domain knowledge and experience design.	Automatically learn feature extraction by training deep learning models (such as CNN).
Model training	No model training is involved, and the feature extraction process is fixed.	A large amount of data is required to train the model to recognize and learn the feature representation in the image automatically.
Feature expression ability	Limited by human domain knowledge and experience, it may be impossible to capture all the details in the image fully.	It can automatically identify and express richer and more abstract image features through hierarchical learning.
Generalization	May have limited generalization capabilities across different tasks and datasets	It has strong generalization ability, especially when dealing with large-scale and complex data sets.

## 4 Outlook

With the advancement of nuclear medicine technology, many studies have shown that dual-modality PET/CT radiomics models outperform models using CT or PET alone in the qualitative diagnosis of pulmonary nodules (PNs) (10, 11). Techniques such as dual-tracer imaging (e.g., using ^18^F-FLT and ^18^F-FDG) (37) and dual-time-point imaging (DTPI) (22, 38) have also significantly improved diagnostic accuracy.

Data imbalance is a major challenge in radiomics research for diagnosing benign and malignant pulmonary nodules (PNs), leading to biased diagnostic results. Several strategies can be employed to address this issue. First, data augmentation can increase the diversity and quantity of samples. Second, resampling or oversampling at the feature level: for example, using SMOTE (Synthetic Minority Over-sampling Technique) (53) to generate synthetic samples can help avoid model overfitting. Lastly, semi-supervised learning methods can be used: for example, pseudo-labeling, which involves vote-based classification of unlabeled samples, can improve model robustness. Additionally, Generative Adversarial Networks (GANs) have shown significant effectiveness in generating high-fidelity synthetic data (54). For instance, GAN-based methods have successfully synthesized high-quality PET images from diagnostic CT scans, achieving quality and tumor contrast comparable to actual PET images (55). Applying these emerging methods in medical image analysis has already seen some success (56–58).

Most radiomics studies still rely on single-center and small-scale retrospective research, lacking prospective studies and external independent validation. This limitation results in models with constrained generalization ability. To improve these models’ performance and clinical applicability, future efforts should focus on establishing large databases through multi-center collaborations and using external validation sets to evaluate the models. This approach will enhance the models’ generalization ability and reliability.

With the rapid development of radiomics and deep learning technologies, the role of PET/CT radiomics and deep learning in clinical practice is increasingly prominent. Through the in-depth mining and accurate analysis of massive imaging data, it can generate more valuable non-invasive diagnostic indicators. This not only enables patients to avoid the pain and risks brought by unnecessary biopsies but also significantly reduces the incidence of biopsy-related complications. Meanwhile, it provides a reliable basis for clinical treatment decisions, helping patients obtain accurate diagnosis and timely treatment in the early stage of the disease, thus effectively improving the cure rate. In addition, it can also reduce unnecessary repeated examinations and long-term follow-up observations. In conclusion, PET/CT radiomics and deep learning show broad clinical application prospects in pulmonary nodule diagnosis and have important value that cannot be ignored.

## 5 Conclusion

PET/CT radiomics has significantly improved the accuracy of diagnosing the benign or malignant nature of pulmonary nodules (PNs) by deeply exploring the vast amount of hidden information in conventional imaging. However, issues related to the availability of technology, the reproducibility of results, and the robustness and precision of features still limit its widespread clinical application. As multi-center research progresses, large-scale datasets are established, and new technologies continue to develop, radiomics is expected to play an increasingly important role in diagnosing PNs, providing strong support for clinical decision-making.
